# Trace metals contamination in freshwater fish in Brazil: related factors, environmental impacts, and risks to human health

**DOI:** 10.1007/s10661-026-15977-9

**Published:** 2026-10-08

**Authors:** Danielma Silva Maia, Jordi Sierra, Esther Martí, Guilherme Sgobbi Zagui

**Affiliations:** 1 Postgraduate Program in Environmental Technology, University of Ribeirão Preto, Ribeirão Preto, Brazil; 2 Faculty of Pharmacy and Food Sciences, University of Barcelona, Catalonia, Spain; 3 Department of Medicine, University of Ribeirão Preto, Ribeirão Preto, Brazil

**Keywords:** Trace metals, Freshwater fish, Anthropogenic impact, Food safety, Risk assessment

## Abstract

Freshwater fish are essential components of the aquatic food chain and an important source of protein and nutrients for various communities, especially riverine populations. However, water pollution is one of the main causes of metal bioaccumulation in the organs of these animals. This study conducted a systematic review of the scientific literature on trace metal contamination in freshwater fish in Brazil. The methodological procedure was structured in three stages: definition of the research problem, application of a relevance test with inclusion and exclusion criteria, and selection of eligible studies for the review. Regional heterogeneity was observed in the scientific production on metals in freshwater fish in Brazil, with a higher concentration of studies in the Northern region. The studies indicate a predominance of native species and demonstrate that trophic level, feeding habits, and interaction with the habitat influence the bioaccumulation of metals in freshwater fish in Brazil. There was also consensus that the metals of greatest toxicological and environmental relevance are Pb, Cd, Cr, and Hg, which were the most frequently identified in the studies. The contamination profile of metals in fish varies regionally and is directly related to anthropogenic activities such as mining, intensive agriculture, urbanization, and effluent discharge. Health risk assessment studies indicate that the daily intake of some potentially toxic trace metals (cadmium, lead, and mercury) may exceed the maximum limits established by legislation, mainly affecting populations with high fish consumption, such as Amazonian riverine and Indigenous communities.

**Supplementary Information:**

The online version contains supplementary material available at https://doi.org/10.1007/s10661-026-15977-9.

## Introduction

The aquatic environment is essential for the conservation of biodiversity, the supply of water to the population, and socioeconomic development, especially in countries with abundant water resources, such as Brazil. The country holds about 12% of the planet’s freshwater, with an extensive network of river basins, rivers, and aquifers that play a strategic role in the global availability of water resources (BRASIL, 2025). However, water pollution has intensified with the growth of the global human population, since human practices (domestic, industrial, or commercial) generate waste that reaches water bodies, causing significant implications (Baranosch et al., 2024). Similarly, Silva et al. (2023a, 2023b) state that aquatic environments are constantly affected by pollution caused by human activities that generate effluents containing various contaminants, including potentially toxic metals.

Freshwater fish represent one of the main components of the aquatic food chain and constitute an important source of protein and micronutrients for many communities, including riverside and traditional populations. According to the Food and Agriculture Organization of the United Nations (FAO, 2024), the global average fish consumption is estimated at approximately 20.5 kg per person per year. In Brazil, although there are no precise national data for this consumption, studies conducted in riverine communities indicate that this value may be considerably higher. Particularly in the Amazon region, fish consumption can range from 100 to 550 g per person per day and reaches approximately 148 kg per capita per year (Begossi et al., 2019; Dias et al., 2023). Water contaminated by different anthropogenic sources is one of the main causes of metal bioaccumulation in fish organs (Silva et al., 2019).

According to Almashhadany et al. (2025), metals may gradually accumulate in fish tissues over time through a process known as bioaccumulation. In contrast, biomagnification refers to the dietary transfer and progressive accumulation of contaminants across successive trophic levels within the food web (Sterling, 1994; Barron, 2003*apud* Gomes et al., 2021). In this context, Viana et al. (2023) emphasized that the presence of contaminants in these organisms represents a significant concern, as the bioaccumulation of metals in the muscle tissues of fish inhabiting contaminated aquatic environments may adversely affect not only the fish themselves but also other organisms across the food web, including humans, through the trophic transfer of bioaccumulated metals.

Among Brazilian regions, the diversity of river basins and their environmental characteristics may favor the occurrence of different patterns of metal contamination associated with geological, hydrological, and economic particularities in each locality. Azevedo-Silva et al. (2023a, 2023b) highlight that environmental peculiarities are determinants of trace element dynamics and of the study of complex ecosystems. In this context, Santos et al. (2023) reinforce the need to consider the specific geological and physicochemical characteristics of each region, given Brazil’s continental dimension and its great geomorphological diversity. Despite the growing number of studies on this topic, there are still gaps in integrating the available information, which makes it difficult to obtain a broader understanding of the extent and distribution of metal contamination in freshwater fish in the country. Given this scenario, the present study aims to conduct a systematic review of the scientific literature on trace metal contamination in freshwater fish in Brazil, seeking to identify the main metals investigated, the most studied species, the environmental factors associated with contamination, and the potential environmental impacts and risks to human health resulting from the consumption of these organisms.

## Methodology

The present study identified, selected, and critically analyzed scientific studies related to trace metal contamination in freshwater fish in Brazil. The systematic search process was conducted in January 2026 and involved the screening and eligibility assessment of bibliographic articles. The methodological procedure was organized into three phases. First, the research problem was defined. Next, a relevance test was applied, which allowed the establishment of the inclusion and exclusion criteria. Finally, the eligible studies were identified and selected for inclusion in the review.

In order to guide the development of this systematic review, the following research question was established based on PICO strategy (Guyatt et al., 2008): “What are the levels of trace metal contamination in freshwater fish in Brazil, and what are the environmental implications and human health risks associated with the consumption of these organisms?”. For the selection of articles, inclusion criteria defined in the relevance test were applied, namely: (a) studies addressing metal contamination in freshwater fish; (b) studies presenting relevant data on bioaccumulation and biomagnification processes; (c) studies involving environmental implications and/or human health risks associated with the consumption of contaminated fish; and (d) studies published between 2020 and 2025.

The search for articles was conducted in the following electronic databases: PubMed, Scopus, SciELO and Web of Science. The search strategy was based on the combination of terms related to fish studies, the type of contaminant, the ecotoxicological processes involved, and the geographic area of interest. The following descriptors and their equivalents in Brazilian Portuguese and English were employed: fish, heavy metals, pollution, bioaccumulation, biomagnification, contamination, and Brazil. The terms were combined using Boolean operators (AND/OR) and applied to the title, abstract, and keyword fields, according to the specificities of each database.

**Fig. 1:**
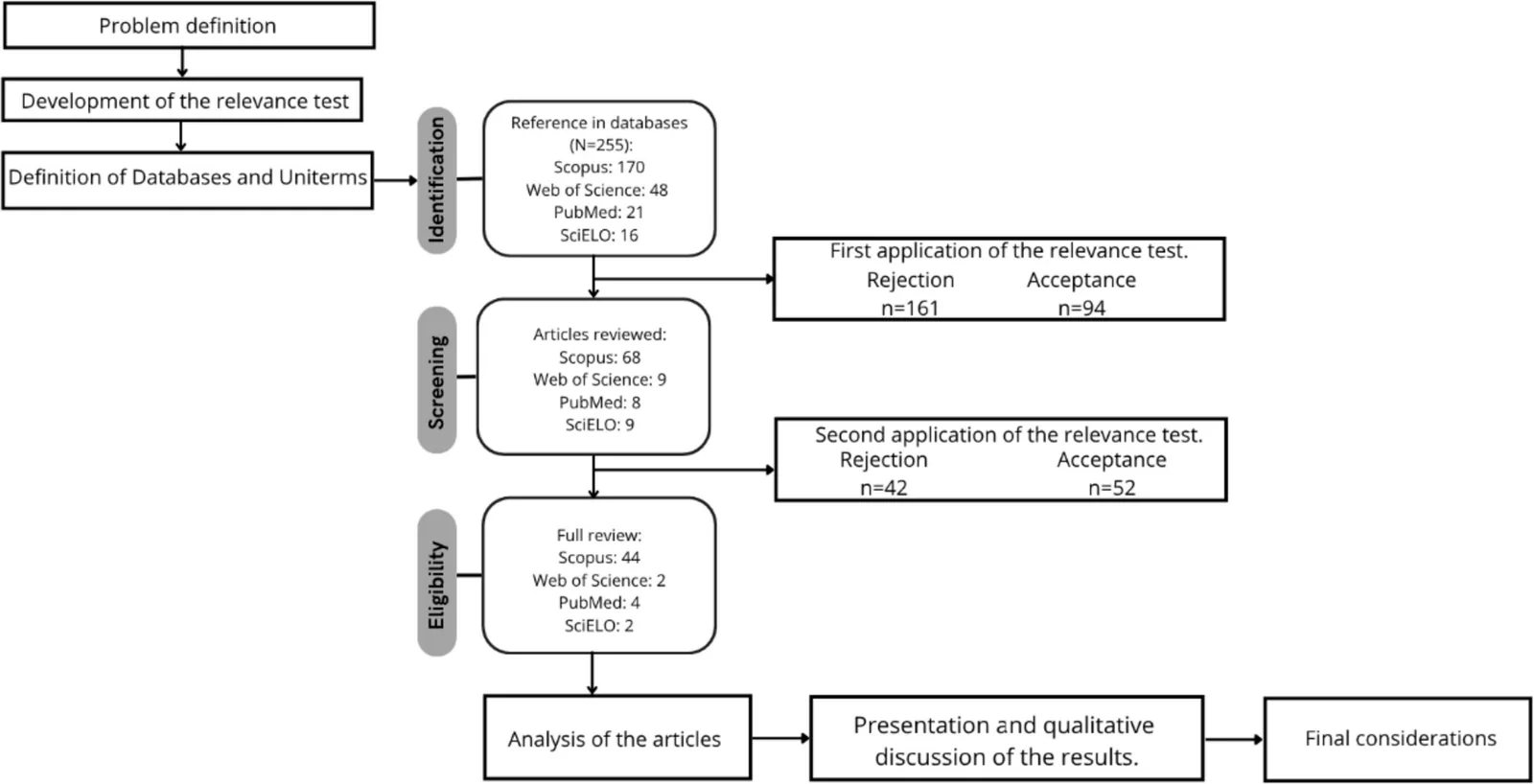
PRISMA flowchart of the systematic review process

The study selection process was summarized using the PRISMA (Preferred Reporting Items for Systematic Reviews and Meta-Analyses) flow diagram, which included duplicate removal, title and abstract screening, and subsequent full-text assessment, as illustrated in Fig. 1. Initially, 255 articles were identified through searches of the selected databases. The retrieved records were then assessed according to the predefined inclusion and exclusion criteria. At this stage, studies published outside the established time frame, investigations addressing metal contamination in marine fish, duplicate records, and studies that did not meet the objectives of this review were excluded. During this preliminary screening process, 161 studies were excluded, and 94 articles were selected for abstract screening. Of these, 52 studies met the eligibility criteria and were included in the full-text analysis.

## Results and discussion

### Geographic distribution of studies

**Fig. 2:**
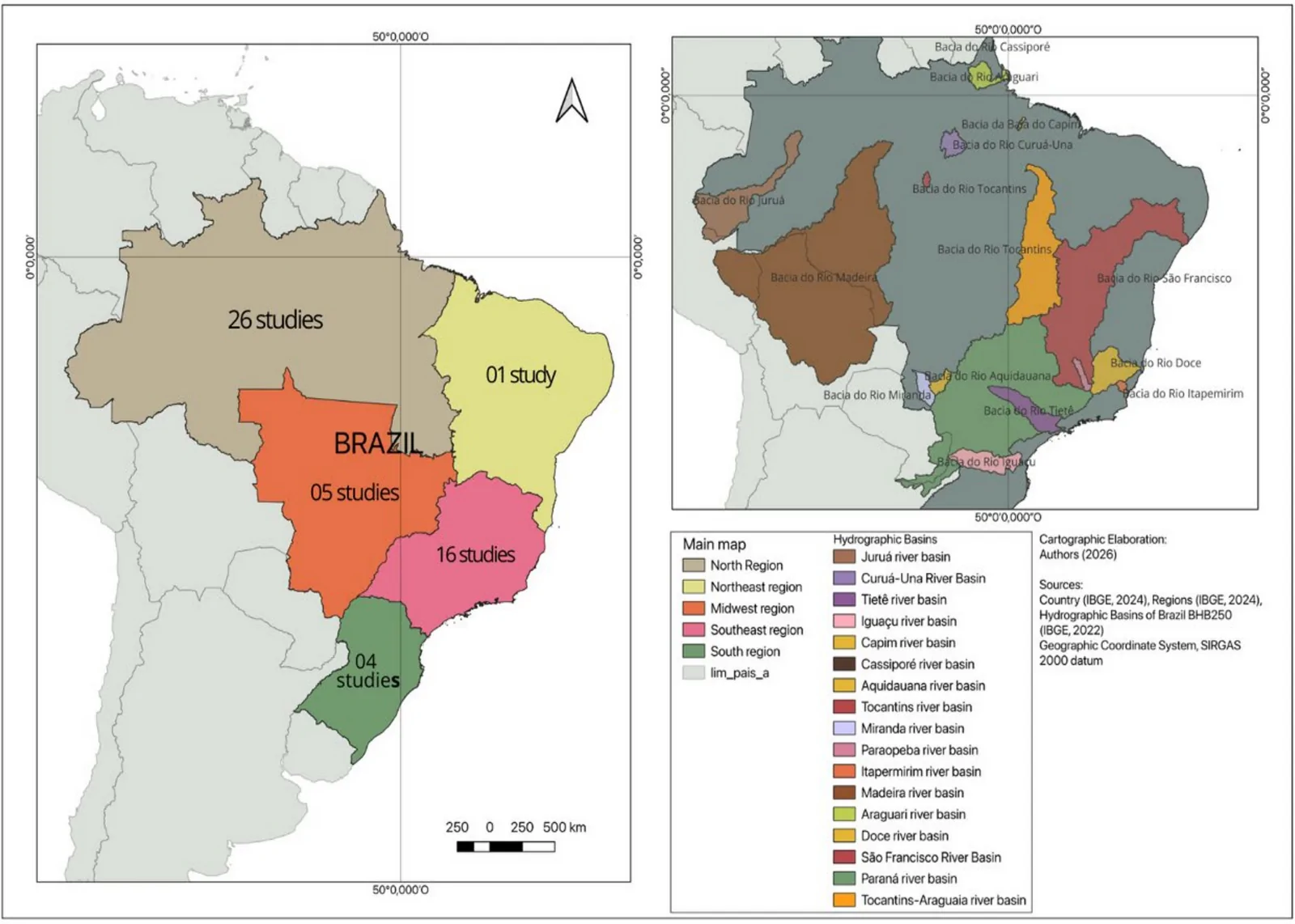
Geographic distribution of studies found on trace metal contamination in freshwater fish in Brazil

The geographic distribution of the analyzed studies, as well as the main river basins investigated, is summarized in Fig. 2. The territorial division of Brazil into regions with distinct environmental and hydrological characteristics is associated with a wide diversity of river basins and river types, which directly influence the bioaccumulation of metals in these water resources. In this context, a regional heterogeneity was observed in Brazilian scientific production on metal contamination in freshwater fish, with a significant concentration of studies in certain regions of the country. This fact suggests that the distribution of studies does not occur uniformly, being concentrated in areas that present greater socio-environmental interactions between the population and water resources, or that have a relevant history of environmental impacts associated with predominant anthropogenic and economic activities. In addition, the concentration of research in some areas to the detriment of others may indicate gaps in scientific knowledge and may limit the monitoring of contaminants in less investigated regions.

The North Region showed the highest representation, with 26 studies, mainly concentrated in the states of Pará and Amazonas. Most of these studies address mercury contamination, often associated with mining activities. The research was conducted in the Amazon Basin, which is composed of several sub-basins, including analyses carried out in regional urban centers, aquaculture systems, and natural environments. Among the main sub-basins studied are the Madeira, Araguari, Juruá, Tocantins, Curuá-Una, Capim, and Cassiporé river basins, encompassing studies on bioaccumulation, biomagnification, and environmental monitoring. The high frequency of studies in this region highlights environmental and social concerns regarding contamination of Amazonian water resources and its potential impacts on riverside populations, since fish is one of the main food sources for local inhabitants. In this regard, Silva and Lima (2020) emphasize that fish has great socioeconomic, cultural, and nutritional importance for Amazonian populations.

The Southeast Region was the second most represented, with 16 studies, mainly covering the states of Minas Gerais, Espírito Santo, São Paulo, and Rio de Janeiro. In this region, there is a predominance of research involving the Doce River Basin, which has been widely investigated following the collapse of the Fundão dam, where studies highlight environmental impacts resulting from mining tailings. The sources consulted also emphasize the Paraopeba River Basin, analyzed after the Brumadinho disaster, as well as the São Francisco River Basin, the Itapemirim River Basin, and the Tietê Basin. In the Central-West Region, 5 studies were identified, with research mainly focusing on the assessment of environmental impacts associated with anthropogenic activities, highlighting the Pantanal as an ecosystem highly vulnerable to the introduction of contaminants. In this region, the studies were concentrated in the Paraguay River Basin, including the Miranda and Aquidauana rivers, and in the Paraná River Basin, with investigations on bioindicators and the impact of land use. The Tocantins–Araguaia River Basin, which involves states from other regions, was also analyzed regarding mercury biomagnification and its relationship with the trophic position of species.

The Southern Region presented 4 studies, two conducted in the state of Paraná, one in Santa Catarina, and one in Rio Grande do Sul, in which the studies focus on the Iguaçu River basin and the Tramandaí River. These sources investigated the presence of metals in freshwater fish mainly associated with agricultural and industrial activities, as well as land use. Finally, the Northeast Region was the least represented, with only one study found, focused on the evaluation of water quality and metal contamination in fish in the estuary of the Perizes River, in the state of Maranhão. It is important to note that some of the studies included in this review adopted a multiregional approach, as they encompassed river basins extending across more than one Brazilian state. This characteristic reflects the fact that river basins are natural systems that frequently transcend state boundaries. For example, Quintela et al. (2024) investigated the Upper Paraguay River Basin (Pantanal), which is shared by the states of Mato Grosso and Mato Grosso do Sul. Custódio et al. (2020) analyzed samples collected simultaneously in the states of Minas Gerais (Southeast Region) and Pará (North Region), while Moraes et al. (2023) evaluated the Tocantins–Araguaia River Basin, which covers areas of both the Central-West and North regions. Similarly, Paiva et al. (2024) conducted their study in the Tucuruí Reservoir, located in the state of Pará, within the Tocantins–Araguaia River Basin, a hydrographic system primarily fed by the Tocantins, Araguaia, and Itacaiúnas rivers. Therefore, the classification of these studies as multiregional reflects the spatial extent of the river basins investigated.

### Most studied contaminated fish species in Brazil

The selected studies predominantly investigated native fish species, with a clear classification according to trophic level, highlighting the importance of ecological position, feeding habits, and habitat use in shaping patterns of metal bioaccumulation in Brazilian freshwater fish. Although trophic-level classification also enables the assessment of biomagnification, this process is not necessarily associated with bioaccumulation. In some cases, trophic biodilution has been reported, whereby contaminant concentrations decrease with increasing trophic level, resulting in top predators exhibiting the lowest concentrations (Azevedo-Silva et al., 2023a, 2023b). Among potentially toxic elements, only mercury (Hg) shows consistent evidence of biomagnification across different ecosystems (Campbell et al., 2008; Clayden et al., 2015; Lavoie et al., 2013). In contrast, this phenomenon is less common for other elements, whose concentrations tend to remain independent of trophic level or even decrease along the food web.

**Fig. 3:**
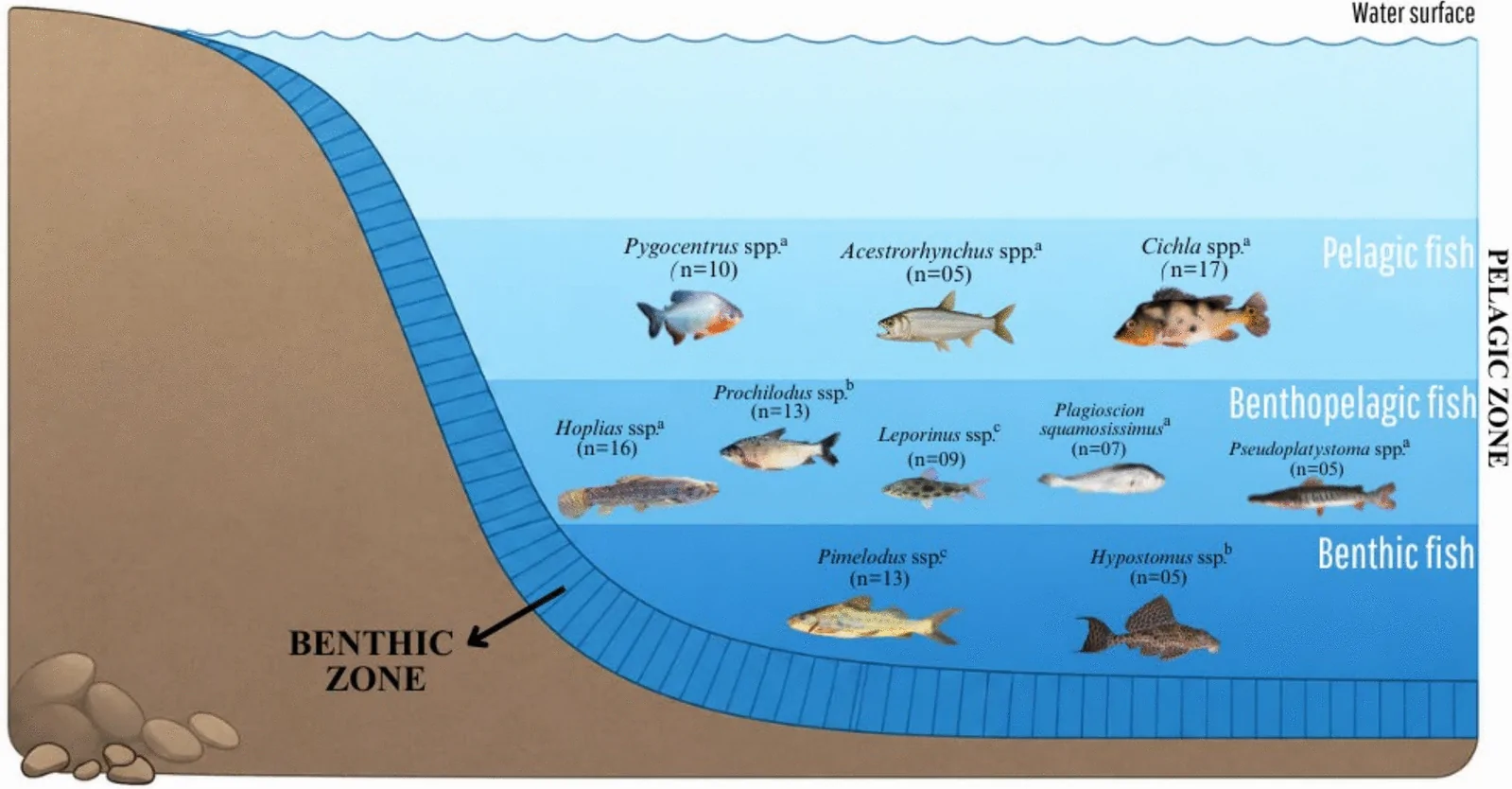
Schematic representation of the distribution of freshwater fish along the water column, eating habits (**a**: carnivore, **b**: detritivore, and **c**: omnivorous), and the number of studies identified (n)

Trace element concentrations in fish can be influenced both by their bioavailability in the aquatic environment and by the foraging behavior of different species. Viana et al. (2022) reported that differences in exposure to inorganic contaminants among fish species are closely associated with feeding and foraging strategies, as species occupy different trophic levels and the bioavailability of inorganic elements varies across aquatic environments. In this context, the bioavailability of trace metals in the aquatic environment is an important factor in determining fish exposure to inorganic contaminants, as their availability for uptake by organisms may vary according to the physicochemical characteristics of the environment and the chemical form in which these elements occur. Fortes et al. (2023) further emphasize that metal bioaccumulation/bioconcentration in fish is a complex process that depends on the bioavailability of metals in the water and the species-specific physiology of each fish; therefore, the availability of metals in the aquatic environment is, in turn, influenced by physicochemical variables such as alkalinity, temperature, and pH. Regarding foraging behavior and species distribution within the water column, factors that may also significantly influence fish exposure to contaminants present in the aquatic environment, Lacerda et al. (2020) reported that benthic species which maintain close contact with sediments generally accumulate higher concentrations of pollutants than pelagic species. This occurs because the latter are predominantly exposed to contaminants present in the water column, where their concentrations are typically lower than those found in sediments. Figure 3 and Table S1 present the freshwater fish species most frequently reported in the selected studies and, consequently, the most extensively investigated, together with their respective feeding habits and positions within the water column.

According to Bevitório et al. (2022), the study of different species makes it possible to understand bioaccumulation, since omnivorous, herbivorous, and carnivorous fish assimilate different amounts of metals from their diet. Based on the analysis of the sources identified, there was a predominance of studies focused on carnivorous and/or piscivorous species, particularly *Cichla* spp., *Hoplias* spp., and *Pygocentrus* spp. Carnivorous/piscivorous species occupy higher positions in the food chain and have a high potential for biomagnification due to their predatory behavior, often associated with larger body size in some species, which favors the accumulation of metals over time. According to Paschoalini and Bazzoli (2021), top predators tend to accumulate greater amounts of metals compared with primary consumers because of their high intake of contaminated organisms. The study by Albuquerque et al. (2020) confirms that large carnivorous species at the top of the food chain accumulate significantly higher levels of mercury (Hg) than smaller herbivorous and detritivorous fish species. Meschede et al. (2024), when comparing Hg concentrations in fish according to feeding habits, found significantly higher levels in carnivorous species (*Arapaima* sp.*, Brachyplatystoma filamentosum, Pseudoplatystoma fasciatumz,* and *Cichla monoculus*).

Detritivorous species, particularly those of the genus *Prochilodus* spp., popularly known as curimbatá or curimatã, also showed high representation among the analyzed studies. Studies by Santos et al. (2020a, 2020b), Waichman et al. (2025), and Viana et al. (2022) reported metal concentrations in this genus above the limits established by health regulatory agencies. This occurs because the bioaccumulation of metals in detritivorous species is directly related to their feeding behavior, as these fish typically forage in benthic environments, feeding on organic detritus and particles associated with sediments. Benthic fish species with detritivorous feeding habits tend to accumulate greater loads of chemical contaminants because they remain in constant contact with sediments and riverbed residues while feeding (Lacerda et al., 2020). This occurs because river sediments act as reservoirs for numerous chemical contaminants (Viana et al., 2022). In other words, benthic detritivorous species tend to present greater exposure and, consequently, a higher potential for the bioaccumulation of these contaminants.

The *Pimelodus maculatus* (mandi) and *Leporinus* spp. (piau) species, which have omnivorous feeding habits, were also frequently addressed in the literature. Although omnivorous fish generally occupy intermediate trophic levels, their feeding habits may lead to significant bioaccumulation rates, especially when they consume benthic organisms or contaminated sediments. In the study conducted by Santos et al. (2020a, 2020b), when evaluating the species *Pimelodus maculatus* and *Leporinus* spp., they identified high concentrations of some metals in the muscle and liver of these fish, showing significantly higher levels compared to other species. Similar results were described in omnivorous fish by Quintela et al. (2024) and Souza et al. (2025) that found high mean and individual values for certain metals, in some cases exceeding the maximum limits established for human consumption.

Regarding position in the water column, the analyzed studies predominantly focused on pelagic species, especially carnivorous and omnivorous ones. Secondarily, the bibliographic sources addressed species with benthic foraging habits, that is, species that explore river substrates, typically associated with detritivorous fish and some omnivores. In contrast, benthopelagic predatory species were less represented in the consulted sources. The analyzed sources also indicated that metal concentrations in fish vary significantly among different organs, being influenced by the physiological function of each tissue. According to Leite et al. (2021), the main routes of metal absorption in fish are the gills, through which metals enter the circulatory system, reach the liver, and, through bile, are transported to the intestine. Metabolically more active organs, such as the liver, gills, and kidneys of teleost fish, accumulate significantly higher levels of trace metals compared with less active tissues, such as vertebrate muscle (Jerônimo et al., 2023; Kalantzi et al., 2013; Porto et al., 2024). Some authors found higher concentrations of metals in the liver compared with muscle (Leite et al., 2021; Silva and Lima, 2020; dos Santos et al., 2020a, 2020b).

### Metals analyzed and their concentration in freshwater fish in Brazil

Based on the evaluation of the selected studies, it was found that the most investigated metals vary according to the geographic region and the objectives of each study. However, there was a consensus regarding the chemical elements considered of greatest toxicological and environmental relevance, with lead (Pb), cadmium (Cd), and chromium (Cr) being the most frequently identified in most of the sources analyzed. Mercury (Hg) also showed high frequency, being mentioned individually or highlighted in several studies, particularly in research conducted in the Amazon region. Less frequently, some studies also investigated trace elements and other metals, especially iron (Fe), manganese (Mn), copper (Cu), and zinc (Zn).

In Brazil, the National Health Surveillance Agency (ANVISA) establishes maximum tolerated limits for various contaminants present in foods, including fish, among these contaminants, trace elements such as mercury (Hg), lead (Pb), cadmium (Cd), and arsenic (As) stand out, with reference values defined in Normative Instruction N°. 160 (2022). To provide an international benchmark for these safety standards, it is noteworthy that ANVISA’s limits are equivalent to those established in the European Union (Regulations (EU) 2023/915 and (EU) 2025/1891). However, for chromium (Cr), this agency does not establish a safety limit for foods, defining only reference doses for an intake considered safe, which ranges between 22.5 and 45 µg/day. In turn, international agencies indicate that safety limits for chromium intake vary according to its chemical form, and, in general, no specific values are defined for the total dietary intake of this element. Therefore, the priority metals in Brazilian freshwater environments, according to the studies analyzed, and due to their toxicity, frequency of occurrence above legal limits, and potential for bioaccumulation, are mercury (Hg), lead (Pb), cadmium (Cd), chromium (Cr), and arsenic (As), with the first four classified as heavy metals and the latter as a metalloid (Albuquerque et al., 2020; Costa et al., 2022; Gomes et al., 2021). Table 1 presents the main results of the studies analyzed, highlighting the concentrations of Hg, Pb, and Cd determined in the muscle of some fish species, including individual values that exceeded the maximum tolerated limits established by ANVISA.

**Table 1:** Concentrations of trace metals in freshwater fish above safety limits

Trace metals and maximum tolerated limits (ANVISA, 2022)	Fish species	Sample size (*n*)	Metal levels (mg/kg)	Descriptive statistic	References
Total mercury (Hg) 0.5 mg/kg (non-predatory fish) and 1.0 mg/kg (predatory fish)	*Hoplias* ssp.^a^	8	0.94	Mean	Silva and Lima (2020)
	*Pseudoplatystomas* ssp.ª	10	0.78	Mean	
	*Plagioscion squamosissimusª*	9	0.78	Mean	
	*Ageneiosus* ssp.ª	5	0.52	Mean	
	*Cichla* spp.ª	6	0.60	Mean	
	*Plagioscion squamosissimus*ª	120	1.09 ± 0.71	Mean ± SD	Costa et al. (2022)
	*Serrasalmus brandtii*ª	178	1.63	Max. conc	Gomes et al. (2021)
	*Pygocentrus piraya*ª	15	1.20	Max. conc	
	Acestrorhynchus falcirostrisª	5	1.16	Max. conc	Brito et al. (2025)
	*Ageneiosus dentatus*ª	6	1.08	Max. conc	
	*Ilisha amazonica*^*b*^	6	0.71	Max. conc	
Lead (Pb) 0.3 mg/kg (fish and fishery products)	*Salminus franciscanusª*	18	2.11 ± 0.16	Mean ± SD	Savassi et al. (2020)
	*Prochilodus* ssp.^c^	7	0.47 ± 0.12	Mean ± SD	Viana et al. (2022)
	*Hypostomus* ssp.^c^	5	0.34 ± 0.03	Mean ± SD	
	*Brycon hilarii*^c^	11	0.40 ± 0.07	Mean ± SD	
	*Mylossoma duriventre*^*d*^	4	0.33 ± 0.01	Mean ± SD	
	Leporinus fasciatus^e^	6	0.40 ± 0.55	Mean ± SD	Viana et al. (2023)
	*Triportheus auritus*^e^	5	0.70 ± 0.67	Mean ± SD	
	*Curimata incompta*^c^	28	0.81 ± 0.62	Mean ± SD	
	*Hemiodus unimaculatus*^c^	40	0.44 ± 0.52	Mean ± SD	
Cadmium (Cd) 0.05 mg/kg to 0.1 mg/kg (varies depending on the type of fish)	*Ageneiosus nassaª*	3	0.09	-	Souza et al. (2025)
	*Acestrorhynchus altusª*	5	0.07	-	
	*Leporinus friderici*^*e*^	9	0.07	-	
	*Serrasalmus spilopleuraª*	4	0.07	-	
	*Pimelodus nattereri*^*e*^	5	0.07	-	
	*Cichla amazonarumª*	7	0.06	-	
	*Krobia guianensisª*	20	0.06	-	
	*Astyanax lacustris*^*e*^	22	0.06	-	
	*Mylossoma rubripinnis*^*c*^	10	0.06	-	
	*Oreochromis niloticus*^*e*^	7	0.05	-	
	*Hoplias malabaricusª*	4	0.06 ± 0.00	Mean ± SD	Viana et al. (2023)
	*Cichla pinimaª*	6	0.06 ± 0.00	Mean ± SD	
	*Leporinus fasciatus*^*e*^	6	0.05 ± 0.34	Mean ± SD	
	*Gasteropelecus proximusª*	16	0.06 ± 0.00	Mean ± SD	
	*Serrasalmus jurupariª*	4	0.06 ± 0.00	Mean ± SD	
	*Triportheus auritus*^*e*^	5	0.40 ± 0.48	Mean ± SD	
	*Curimata incompta*^*c*^	28	0.47 ± 0.44	Mean ± SD	
	*Hemiodus unimaculatus*^*c*^	40	0.23 ± 0.35	Mean ± SD	
	*Salminus franciscanusª*	18	0.33 ± 0.02	Mean ± SD	Savassi et al. (2020)
	*Prochilodus* ssp*.*^c^	7	0.28 ± 0.04	Mean ± SD	Viana et al. (2022)
	*Hypostomus* ssp*.*^c^	5	0.22 ± 0.01	Mean ± SD	
	*Brycon hilarii*^*e*^	11	0.24 ± 0.03	Mean ± SD	
	*Mylossoma duriventre*^*d*^	4	0.21 ± 0.02	Mean ± SD	

*Max. conc.* maximum concentration (highest concentration observed among the samples analyzed in the respective study)

^a^Carnivore

^b^Planctivoro

^c^Detritivore

^d^Herbivore

^e^Omnivorous

Mercury is recognized for its high potential for toxicity and bioaccumulation. Methylmercury (MeHg) is the most toxic form of mercury and is generated through the anaerobic microbial methylation of mercury present in sediments (Ullrich et al., 2001; Gomes et al., 2020), where the main route of human exposure to MeHg occurs through fish consumption. In agreement, Basta et al. (2023) report that MeHg is the most dangerous chemical form for human health and for the ecosystem. In this context, it was observed that most available studies have focused on determining total mercury (THg) levels, while MeHg has been significantly less investigated. The study by Meschede et al. (2024) found that the highest Hg levels were detected in carnivorous species, with statistically significant differences compared to other trophic levels. Vasconcellos et al. (2021) further emphasize that biomagnification along the trophic chain was confirmed in Indigenous communities of the Tapajós region, where piscivorous fish presented Hg levels about four times higher than non-piscivorous species. According to the data in Table 1, the authors reported that Hg concentrations in fish exceeded the established safety limits. Moreover, Nyholt et al. (2022) observed that 70% of the analyzed samples showed Hg concentrations above the recommended guidelines for consumption. Albuquerque et al. (2021) found high concentrations of mercury (Hg) in the liver of *Cichla* spp., reaching 26.7 mg/kg dry weight. Basta et al. (2023), in their study, identified that 21.3% of the studied species presented Hg levels equal to or higher than the safe limit. Albuquerque et al. (2020) also reported that 16% of the analyzed carnivorous species presented maximum Hg concentrations above the values established by Brazilian legislation.

Lead (Pb) is widely recognized as one of the most toxic metals to living organisms and has no known biological function (Patocka et al., 2018, apud Teixeira et al., 2025). The bioaccumulation and biomagnification of Pb in fish occur through complex processes. According to Azevedo-Silva et al. (2023a, 2023b), lead mimics divalent cations (Ca, Mg, Fe, Zn, and Mn) and is distributed throughout fish tissues by replacing essential elements in membrane transport systems. Some studies indicate that lead has biomagnification potential, with higher concentrations observed in top predators such as carnivorous and piscivorous species (Quintela et al., 2024; Savassi et al., 2020; Waichman et al., 2025). However, other investigations reported trophic dilution, in which concentrations of this metal decreased at higher trophic levels (Azevedo-Meschede et al., 2024; Silva et al., 2023a, 2023b; Viana et al., 2023), suggesting that its trophic transfer does not follow a uniform pattern. In accordance with the data presented in Table 1, other studies also reported safety limits being exceeded in Pb concentrations in fish. Lima et al. (2022), reported Pb concentrations above 2 mg kg⁻^1^, exceeding the limits established by Brazilian legislation for fishery products. Vergílio et al. (2020) found that 41% of fish samples presented concentrations above the established national standards. Santos et al. (2020a, 2020b) observed that Pb levels in the muscle and liver of most analyzed species exceeded the recommended reference values. Additionally, Albuquerque et al. (2023) reported lead concentrations above the maximum permitted limit in most of the collected samples.

Cadmium (Cd) stood out for presenting the highest bioconcentration factor (BCF), with levels in fish up to ten times higher than those found in water. Considering that *Hopilas malabaricus* is carnivorous, this finding suggests possible biomagnification of the metal along the food chain (Leite et al., 2023). Results presented in Table 1 show the concerning presence of Cd in fish, particularly in the studies by Souza et al. (2025), which reported alarming results, with Cd concentrations above the maximum limit permitted by Brazilian legislation in all 10 evaluated species. Viana et al. (2023) identified that 8 of the 11 analyzed species presented Cd levels above the legal limit, with emphasis on concentrations above 0.05 mg/kg. In addition, other studies similarly highlighted this concern in their investigations. Baranoschi et al. (2024), when analyzing endemic species of the genus *Cambeva* spp., observed that most samples showed Cd concentrations above the values permitted by both Brazilian and European legislation. Freitas et al. (2025) also reported an isolated case in which a specimen of tucunaré presented Cd levels in muscle tissue above the maximum permitted limit.

Regarding chromium (Cr), the evaluated studies mainly focus on total chromium; however, chromium toxicity in aquatic ecosystems and for human health is strongly related to its chemical speciation. Cr may be present in the environment as Cr(III) or Cr(VI), the latter being much more soluble in water and more toxic to a wide range of living organisms (Waichman et al., 2025). With respect to its biomagnification potential, several studies have reported a trophic biodilution pattern, whereby concentrations of this metal are higher in fish at lower trophic levels and progressively decline with increasing trophic level, indicating that its trophic transfer does not lead to biomagnification. In accordance, Nascimento et al. (2022) identified a median chromium concentration ranging from 0.07 mg/kg in carnivorous species to 0.62 mg/kg in herbivorous species. In the study by Azevedo-Silva et al. (2023a, 2023b), the results indicated higher mean Cr levels in detritivorous and iliophagous species, which were significantly higher than those found in piscivorous and omnivorous species. Waichman et al. (2025), in a large-scale analysis involving 11 species, reported elevated Cr concentrations in 83% of the samples, with occurrence in all evaluated species. Similarly, the study by Lima et al. (2022) also found elevated Cr levels in all species evaluated. In addition, Savassi et al. (2020) highlighted the spatial variability of Cr pollution, reporting mean concentrations of 1.71 mg/kg in the Paraopeba River compared to 0.55 mg/kg in the Abaeté River. In general, the studies reported Cr concentrations in fish that are potentially concerning from a toxicological perspective. Although no maximum tolerable limit has been established for this matrix, these levels may exceed the safe oral reference dose for humans, depending on the consumption rate.

Considering the reviewed studies, arsenic (As) was often detected at low concentrations, although some investigations reported situations in which its levels exceeded established safety limits. This may be related to the fact that arsenic toxicity and mobility depend on its chemical form, since inorganic As, such as arsenite (AsO_3_^3^⁻) and arsenate (AsO_4_^3^⁻), represent the most toxic and carcinogenic forms. However, the main forms of As in fish are organic arsenic compounds, which are much less toxic (Agency for Toxic Substances and Disease Registry, 2007; Parente et al., 2021). Studies that reported the occurrence of As in fish at concentrations above the safety limits established by Brazilian legislation include the work of Freitas et al. (2025), who recorded a specific case in which a specimen of *pygocentrus nattereri* presented arsenic concentrations in muscle tissue above the maximum limit permitted by current legislation. In addition, Viana et al. (2022) observed that *Prochilodus lineatus* (curimbatá) presented an average arsenic concentration above the standards established by Brazilian legislation.

### Influence of environmental factors on metal pollution patterns

The analyzed studies showed that the distribution and profile of metal contamination in fish present important regional variations, which are directly related to the characteristics of the river basins and the human activities present. Anthropogenic activities, including mining, intensive agricultural practices, urbanization, and the discharge of domestic and industrial effluents, are identified as the main factors associated with metal contamination in water resources. In addition, studies involving areas with hydroelectric dams indicate that these systems act as reservoirs or “sinks” for metals by altering the natural hydrodynamics of rivers and promoting the retention and greater availability of these contaminants in the aquatic environment. In this context, Table 2 presents the main sources of contamination reported in the articles, according to each region.

**Table 2:** Regional patterns, major metals, and sources of contamination in fish from Brazil

Region	Main metals studied	Main sources of contamination	References
North (Amazon)	Hg, Al	Mining; deforestation; geological materials; wildfires and hydroelectric dams	Waichman et al., 2025; Souza et al., 2025; Brito et al., 2025; Meschede et al., 2024; Paiva et al., 2024; Azevedo-Silva et al., 2023a, 2023b; Cantanhêde et al., 2023; Viana et al., 2023; Nyholt et al., 2022; Nascimento et al., 2022; Costa et al., 2022; Felix et al., 2022; Basta et al., 2023; Albuquerque et al., 2020; Silva & Lima, 2020; Albuquerque et al., 2021 e Lima et al., 2022
Southeast	Fe, Mn, Al, Pb, Cr, Cd	Tailings dam failures; mining; industrial and urban effluents; and atmospheric deposition	Freitas et al., 2025; Costa et al., 2022; Matos et al., 2022; Parente et al., 2021; Carvalho et al., 2025; Bevitório et al., 2022; Leite et al., 2023 e Savassi et al., 2020
Central-West (Pantanal and Cerrado)	Cd, Pb, As, Fe, Mn	Agriculture; use of fertilizers and agrochemicals; and mining	Lima et al., 2025; Quintela et al., 2024; Moraes et al., 2023; Viana et al., 2022; Santos et al., 2020a, 2020b
North East	Cr, Pb, Hg	Industrial emissions; refineries; urban wastewater and estuarine activities	Albuquerque et al., 2023
South	Cd, Cu, Fe, Mn	Agriculture; urbanization; domestic wastewater	Baranosch et al., 2024; Teixeira et al., 2025; Rubio-Vargas et al., 2024

Based on the number of studies identified, mercury (Hg) and aluminum (Al) emerged as the main contaminants investigated in the Northern Region of Brazil. Among them, Hg is of particular concern, as its occurrence in the region’s river systems has been extensively documented in the scientific literature and is largely associated with mining activities. Lino et al. (2019), Pestana et al. (2022), Paiva et al. (2024) and Lima et al. (2022) highlighted that the region has a long history of gold mining and deforestation, both of which contribute to elevated Hg concentrations in aquatic ecosystems. Furthermore, Crespo-Lopez et al. (2021) identified forest fires (biomass burning) as the second largest source of mercury emissions in the Amazon region, after artisanal and small-scale gold mining. This process occurs because Amazonian soils are naturally enriched in mercury (Azevedo, 2003; Beckers & Rinklebe, 2017*apud* Crespo-Lopez et al., 2021), while the Amazon rainforest also acts as an atmospheric sink for this metal. During biomass burning, mercury accumulated in vegetation and soils is volatilized, allowing it to be transported over long distances before being redeposited onto soils and aquatic ecosystems. Regarding aluminum, Sparling and Lowe (1996) and Closset et al. (2022) reported that Al is the third most abundant element and the most abundant metallic element in the Earth’s crust, accounting for approximately 8.1% of its mass. However, its concentrations in surface waters can be further increased by anthropogenic activities. Cantanhêde et al. (2023) reported that the Amazon region may naturally exhibit Al concentrations exceeding regulatory limits due to the presence of extensive bauxite deposits, the primary raw material used for aluminum production, which are concentrated mainly in the state of Pará. The predominance of Hg and Al among the elements investigated in the Northern Region highlights the complexity of the issue and reinforces the need for integrated control and monitoring strategies, particularly for Hg, whose high toxicity and environmental persistence have prompted national and international initiatives. From a public policy perspective, the Minamata Convention, enacted in Brazil through Decree Nº. 9,470/2018, aims to protect human health and the environment from anthropogenic emissions and releases of mercury and its compounds. However, efforts to mitigate this threat have not been sufficient to contain the expansion of gold mining in the Brazilian Amazon, particularly over the past 5 years (Basta et al., 2023). Therefore, given the persistence of sources of exposure to these metals, there is a need to strengthen enforcement, environmental monitoring, and measures to protect potentially exposed populations.

In the Southeast Region, contamination patterns are strongly influenced by large-scale mining dam failures and by intense industrial and urban activities. According to Costa et al. (2022), mining tailings dams represent a high environmental risk due to the presence of mining residues such as metals, metalloids, and other trace elements, a risk that becomes even more severe when these dams collapse. The studies reviewed indicate that the tailings disasters in Mariana and Brumadinho represent critical milestones in the massive release of sediments enriched with toxic and non-toxic metals, with direct impacts on the Doce and Paraopeba rivers (Bevitório et al., 2022; Carvalho et al., 2025; Costa et al., 2022; Freitas et al., 2025; Matos et al., 2022; Parente et al., 2021). Additionally, contamination in rivers in State of São Paulo and State of Minas Gerais reinforces the role of industrial effluents, tanneries, and domestic sewage as continuous sources of metals. Leite et al. (2023) identified high Al concentrations in fish from the Jacaré-Pepira and Jacaré-Guaçu rivers (Tietê Basin), associated with the discharge of urban and industrial effluents. Similarly, Savassi et al. (2020), in studies conducted in the Paraopeba River, related contamination to the discharge of domestic, agricultural, mining, and tannery effluents, negatively affecting local fish species. In the State of Espírito Santo, Fortes et al. (2023) point to the contribution of atmospheric particulate matter from steel industry complexes as indirect pathways of contamination, increasing the deposition of certain chemical elements in aquatic systems.

In the Central-West Region, particularly in the Pantanal and the Cerrado, metal contamination is strongly associated with the expansion of agricultural and livestock frontiers. Viana et al. (2022) and Quintela et al. (2024) used fish as bioindicators to assess contamination in Pantanal rivers showing that the region faces pollution threats from human activities such as pesticide use and pasture expansion, which reduce native vegetation, increase erosion, and introduce exotic species, harming aquatic life. In the Northeast and South Regions, the observed patterns predominantly reflect industrial, urban, and agricultural impacts. In the Northeast, high concentrations of some metals in estuaries, such as the Perizes River estuary are associated with the proximity of industrial zones and refineries (Albuquerque et al., 2023). In studies conducted by Baranoschi et al. (2024) and dos Santos et al. (2020a, 2020b), potentially concerning levels of metals in fish were found in agricultural areas.

According to the reviewed articles, several authors also highlighted that seasonal changes and hydrological regimes, resulting from rainfall and river flood periods, are the main factors that mobilize contaminants in water, affecting how metals are transported, their availability, and their bioaccumulation in fish (Cantanhêde et al., 2023; Bevitório et al., 2022; Nascimento et al., 2022; Santos et al., 2023; Nyholt et al., 2022; Brito et al., 2025). In addition, studies were identified that relate metal concentrations to the presence of reservoirs formed by hydroelectric dams. According to Brito et al. (2025), the construction of hydroelectric dams floods extensive forest areas, resulting in the formation of large water reservoirs. This alteration, especially the stratification regime of reservoirs, may favor the accumulation of metals and contribute to exceeding reference limits for the health of fish and humans (Paiva et al., 2024; Willacker et al., 2023), leading to persistent environmental impacts associated with hydroelectric infrastructure.

### Assessment of the risk to human health associated with the consumption of contaminated fish

The consumption of contaminated fish represents a potential risk to human health. According to Hasanein et al. (2022), fish consumption constitutes a significant pathway through which humans are exposed to chemical pollutants, including metals. Similarly, Waichman et al. (2025) reported that individuals who regularly consume contaminated fish may be chronically exposed to these contaminants. This concern is particularly relevant for riverine and Indigenous communities, where fish consumption is substantially higher because it constitutes a major component of the diet and is closely linked to food security, cultural traditions, and economic subsistence. In agreement, Silva and Lima (2020) emphasized the socioeconomic, cultural, and nutritional importance of fish for Amazonian populations, for whom fishing represents the primary source of both income and food. Isaac et al. (2015) reported that the average per capita fish consumption among riverine populations in the Amazon region reaches approximately 462 g of fish per day or 169 kg per year.

Nevertheless, the parameters evaluated in the reviewed studies indicate that the health impacts associated with human exposure to contaminants are influenced by several factors, including the type of metal, the amount ingested, the duration of exposure, and individual susceptibility, with risk varying across population groups, particularly between children and adults. For example, Meschede et al. (2024) assessed non-carcinogenic risks in adults and children and found, based on the hazard quotient (HQ), that mercury (Hg) intake through fish consumption in children reached or exceeded the safety threshold, indicating a potential health risk for this population. Albuquerque et al. (2023) reported that chromium (Cr) concentrations exceeded the maximum limits established by Brazilian legislation in all fish species analyzed, highlighting the considerable health concern for riverine communities that regularly consume locally caught fish due to their prolonged exposure. Similarly, Viana et al. (2022) found that cadmium (Cd) and lead (Pb) concentrations in fish muscle exceeded the maximum permissible limits established by Brazilian regulations for human consumption. Overall, the studies reviewed demonstrated a substantial health risk primarily associated with chronic exposure to non-essential toxic metals, particularly mercury (Hg), and, under specific exposure scenarios, chromium (Cr), lead (Pb), and cadmium (Cd).

In scenarios in which exposed individuals may be subject to non-carcinogenic health risks, the risk ratio expressed by the Hazard Quotient (HQ) is considered, where HQ values >1.0 indicate the possibility of harmful effects, as they represent exposure levels above the limit considered safe. In this regard, Hg was identified as the most critical contaminant due to its high biomagnification potential, associated with the fact that methylmercury (MeHg), its most toxic and dangerous form to humans, represents more than 80% of the total mercury present in fish muscle (Souza-Araujo et al., 2016). In the study conducted by Azevedo-Silva et al. (2023a, 2023b), for non-carcinogenic risk, HQ values were higher than 1.0 for all fish species analyzed in relation to MeHg. However, the study by Lima et al. (2022) reported potentially concerning exposure levels for lead, with HQ values ranging from 1.75 to 3.60 across all fish species evaluated. The Estimated Daily Intake (EDI) is a widely used indicator in food risk assessment studies, allowing the estimation of the amount of a given contaminant ingested daily by an individual through food consumption. The estimated daily intake (EDI) of Hg also exceeded the reference doses (RfD) in almost all Amazonian scenarios. In the study by Meschede et al. (2024), Hg showed EDI estimates higher than the Recommended Daily Intake (RDI) established by ANVISA, involving risks associated with the consumption of four fish species (*Arapaima gigas*, *Brachyplatystoma filamentosum*, *Cichla* sp., and *Pseudoplatystoma fasciatum*) for adults, and five species (*Arapaima gigas*, *Brachyplatystoma filamentosum*, *Pseudoplatystoma fasciatum*, *Cichla ocellaris*, and *Dasyatis pastinaca*) for children.

Although Hg is predominant, other elements also represented risks according to some studies. In the investigation by Waichman et al. (2025), the EDI values for Cr exceeded the reference doses considered safe for almost all analyzed species, in which carcinogenic risks may be expected due to continuous exposure. In the risk assessment carried out by dos Santos et al. (2020a, 2020b), the values obtained for Cd and Pb were higher than 1, indicating carcinogenic risk to human health from the constant consumption of the investigated fish. According to the study by Viana et al. (2023), HQ > 1 were observed in the species *Hoplias malabaricus*, *Leporinus fasciatus*, *Triportheus auritus*, *Curimatella incompta*, *Hemiodus unimaculatus*, and *Plagioscion*, indicating a potential risk to human health, especially considering that these species are widely consumed by local populations. Taken together, these results indicate that the ingestion of potentially toxic metals present in freshwater fish may pose a risk to human health, especially for more vulnerable groups. The occurrence of intake values and risk indices above the established reference limits reinforces the need for continuous monitoring of these contaminants in widely consumed species.

However, it is important to emphasize that the assessment of human health risks should not rely exclusively on the total concentrations of metals determined in fish tissues, since the presence of a contaminant does not necessarily imply its complete availability for absorption by the human body. According to Peijnenburg and Jager (2003), bioavailability and bioaccessibility are important factors in determining whether the concentration of a chemical substance will result in adverse effects in organisms. The authors further highlight that, for a metal to exert systemic toxicity in the human body, it must first be released from the food matrix during gastrointestinal digestion (bioaccessibility) and subsequently cross the intestinal barrier to reach the bloodstream. Therefore, incorporating bioavailability and bioaccessibility parameters into risk assessments may provide a more accurate estimate of human exposure associated with the consumption of contaminated fish, reducing potential overestimations resulting from the exclusive use of total metal concentrations and contributing to a better understanding of the potential health effects associated with the consumption of contaminated fish.

### Research gaps and future perspectives

Despite the large number of studies identified on trace metal contamination in freshwater fish in Brazil, it is important to emphasize that significant scientific gaps still remain, limiting a more comprehensive assessment of environmental and human health risks. In this context, there is a clear need for more detailed investigations focusing on the specific chemical forms of certain metals, such as chromium in its hexavalent form, and especially methylmercury, as well as the inclusion of emerging contaminants, such as inorganic arsenic (As^3+^, As^5+^), which represents the most toxic form. It should be noted that the lack of chemical speciation may lead to an underestimation or imprecision in the assessment of the actual risk.

Considering that fish act as bioindicators of contamination in aquatic ecosystems, future studies should broaden their focus to include physiological and ecological aspects of fish species. Most studies have concentrated on the bioaccumulation of metals in fish organs and tissues; however, sensitive biological systems must also be considered. According to Paschoalini and Bazzoli (2021), the reproductive and nervous systems have rarely been investigated in neotropical fish species over the last decade. Few studies evaluate how cooking processes influence metal concentrations in fish, as most investigations analyze fish only in their fresh state. Recently, Meschede et al. (2024) reported that most of the fish species analyzed showed a tendency toward increased elemental concentrations after cooking, with increases in Al, Cu, and Hg being the most frequently observed. Therefore, the importance of conducting more integrated assessments of risks to human health becomes evident, particularly considering consumption patterns and the characteristics of exposed populations. This issue becomes even more relevant in scenarios where daily fish consumption by adults and children such as in riverine and Indigenous communities is significantly higher, resulting in greater exposure to trace metals. In this regard, the need emerges to discuss the establishment of regional regulatory limits that take into account the specific dietary habits of these populations, in which fish consumption is considerably higher than the national average. Long-term studies are also required to estimate safe intake doses that reflect this particular reality.

Another relevant aspect highlighted by the analyzed studies concerns the influence of land-use and land-cover changes on the dynamics of contamination in aquatic ecosystems. Several studies indicate that anthropogenic interventions, such as the failure of mining tailings dams (Bevitório et al., 2022; Carvalho et al., 2025; Costa et al., 2022; Freitas et al., 2025; Matos et al., 2022) and the construction of hydroelectric dams (Paiva et al., 2024; Brito et al., 2025; Rubio-Vargas et al., 2024), promote significant alterations in the hydrosedimentological dynamics of rivers, potentially increasing the mobilization, redistribution, and bioavailability of metals in aquatic environments. Furthermore, some studies emphasize that modifications in the hydrological cycle, particularly in the flood and drought regimes that characterize the flood pulse, may directly influence contamination levels (Nascimento et al., 2022; Brito et al., 2025; Santos et al., 2023; Nyholt et al., 2022; Albuquerque et al., 2020; Cantanhêde et al., 2023; Parente et al., 2021).

According to Hauser-Davis and Wosnick (2022), increasingly frequent extreme weather events are expected as a result of climate change, which may directly and indirectly affect contaminant dynamics in aquatic ecosystems. In this context, the occurrence of droughts, heavy precipitation and floods, heatwaves, and wildfires may alter the physicochemical conditions of aquatic environments, as well as the mobility, speciation, and bioavailability of trace metals, and some of the studies evaluated in this review have demonstrated this association. Furthermore, additional scientific evidence related to recent extreme events in Brazil raises further concerns regarding these potential effects. Caetano et al. (2025) investigated the presence of metals in water and soil from the Sinos River Basin following the May 2024 floods. Although low concentrations of these elements were detected in the water, significant chromium concentrations were identified in the soil, ranging from 2.400 to 107.560 mg/kg. Johannsson et al. (2026) evaluated water quality and its implications for fish inhabiting the Negro and Solimões rivers during the extreme Amazon drought of 2023 and observed that changes in temperature, dissolved oxygen, pH, conductivity, suspended solids, and dissolved organic carbon may have contributed to increased fish mortality and reduced growth rates. Supporting this evidence, Azevedo et al. (2018) investigated the influence of drought on THg and MeHg bioaccumulation in fish from the lower Paraíba do Sul River basin, southeastern Brazil, and found that contaminant levels were higher during the dry season in most of the species evaluated.

From this perspective, these findings highlight the need for more in-depth investigations addressing the environmental impacts resulting from anthropogenic activities and natural processes, which emerge as key determinants of contamination dynamics. Such processes may intensify metal bioaccumulation in fish and increase the risk of exposure for human populations that depend on these resources as a food source. Future studies should therefore explore in greater detail how land-use changes, extreme events, and alterations in hydrological regimes influence the mobilization, chemical transformation, and bioavailability of these contaminants.

## Conclusion

This systematic review corroborates the critical role of freshwater fish as bioindicators of environmental quality and sentinels of public health risks in Brazil. The scientific literature reveals marked regional heterogeneity across river basins, where metal pollution profiles are shaped by local socio-environmental dynamics and exacerbated by anthropogenic pressures such as mining, agriculture, and urbanization. In this context, it was observed from the consulted sources that the main contamination factors include mining activities, especially in the Amazon, the expansion of agricultural and livestock activities, the discharge of urban and industrial effluents, as well as impacts resulting from mining dams and hydroelectric power plants. Furthermore, natural and hydrological processes, such as seasonal variations, flood regimes, and the physicochemical characteristics of the waters, also play a relevant role in the availability and bioaccumulation of these elements in aquatic resources.

Ecological characteristics, particularly trophic level and foraging behavior, play a fundamental role in determining contaminant burdens in fish. Due to biomagnification, predatory species at higher trophic levels (e.g., carnivorous and piscivorous species) present the highest concentrations of mercury (Hg), posing a severe toxicological risk. On the other hand, detritivorous and omnivorous species tend to bioaccumulate metals associated with sediments such as lead (Pb) and chromium (Cr). Herbivorous fish, which feed on plants, fruits, and seeds, generally show lower Hg concentrations compared with predators. Nevertheless, although herbivorous and detritivorous species are safer in relation to Hg, they may accumulate higher levels of other metals, such as Pb and Cr, due to their foraging habits in riverbed sediments. Therefore, these findings have important public health implications, as the consumption of fish species with a greater potential for bioaccumulation may increase human exposure to potentially toxic metals, thereby elevating the risk of adverse health effects, particularly among riverine and Indigenous communities that rely on fish as their primary source of dietary protein.

Health risk assessments consistently demonstrate that the daily intake of trace elements frequently exceeds the safety limits established by ANVISA, the EU, the USEPA, and the FAO. More concerningly, these standardized limits may be fundamentally insufficient to protect highly vulnerable groups, such as Amazonian riverine and Indigenous communities, whose risk is compounded by a lifetime of exceptionally high fish consumption. In conclusion, most studies warn that fish consumption in several Brazilian regions, especially carnivorous species, should be controlled or limited to prevent damage to public health; in other words, it requires continuous monitoring and guidance on consumption limits.

The analysis of the reviewed studies highlights significant scientific advances on the topic; however, important gaps still remain. Among them are the limited differentiation between the chemical forms of metals, the scarcity of investigations evaluating fish samples after cooking processes, and the insufficient number of studies that consider regional specificities and different dietary consumption patterns. It is also essential to promote the development of regulatory public policies and more efficient management strategies capable of strengthening food safety related to fish consumption and promoting the protection of river basins, thereby contributing to the maintenance of environmental balance.

## Supplementary Information

Below is the link to the electronic supplementary material.

ESM 1 (DOCX 17.4 KB)

## Data Availability

No datasets were generated or analyzed during the current study.
