# Supplementary material for: Trace metals contamination in freshwater fish in Brazil: related factors, environmental impacts, and risks to human health
Source: Environ Monit Assess. 2026 Oct 8;198(11):1163. doi: 10.1007/s10661-026-15977-9 (PMC13649874; doi:10.1007/s10661-026-15977-9)
Supplement: Supplementary file 1 [file 10661_2026_15977_MOESM1_ESM.docx]

**SUPLEMENTAR MATERIAL**

**Trace metals contamination in freshwater fish in Brazil: related factors, environmental impacts, and risks to human health**

Danielma Silva Maia^1^, Jordi Sierra^2^, Esther Martí^2^, Guilherme Sgobbi Zagui^1,3*^

*^1^ Postgraduate Program in Environmental Technology, University of Ribeirão Preto, Brazil.*

*^2^ Faculty of Pharmacy and Food Sciences, University of Barcelona, Catalonia, Spain.*

*^3^ Department of Medicine, University of Ribeirão Preto, Brazil.*

**Corresponding author. E-mail address:* [*gszagui@unaerp.br*](mailto:gszagui@unaerp.br) *(G. S. Zagui).*

**Table S1.** Predominant freshwater fish species in the reviewed studies.

| **Scientific name**  **(common name)** | **Eating habits** | **Position in the water column** | **Number of studies** | **Reference** |
| --- | --- | --- | --- | --- |
| *Cichla* spp.  (Tucunaré) | Carnivore/  piscivore | Pelagic | 17 | Azevedo-Silva et al. (2023); Nascimento et al. (2022); Nyholt et al. (2022); Basta et al. (2023); Waichman et al. (2025); Albuquerque et al. (2020); Meschede et al. (2024); da Silva e Lima (2020); Gomes et al. (2021); Custódio et al. (2020); Brito et al. (2024); Waichman et al. (2025); Viana et al. (2022); Costa et al. (2022); Matos et al.(2022); Gomes et al. (2020); Vasconcelos et al. (2021). |
| *Hoplias* ssp*.*  (Traíra) | Carnivore/  piscivore | Bentopelagic | 15 | Azevedo-Silva et al. (2023); Waichman et al. (2025); Viana et al. (2022); Freitas et al. (2025); Matos et al.(2022); Brito et al. (2024); Custódio et al. (2020); Da Silva e Lima (2020); da Silva et al (2020); Leite et al (2023): Nyholt et al. (2022); Basta et al. (2023); Viana et al. (2022); Vargas et al (2024); Tesser et al. (2021). |
| *Prochilodus* spp.  (Curimbatá) | Detritivore | Bentopelagic | 13 | Nascimento et al. (2022); Santos et al. (2020); Viana et al. (2022); Meschede et al. (2024); Waichman et al. (2025); Freitas et al. (2025); Costa et al.(2022); Custódio et al. (2020); Da Silva e Lima (2020); Nyholt et al. (2022); Silva et al (2020); Vasconcelos et al. (2021); Gomes et al. (2020). |
| *Pimelodus* ssp.  (Mandi) | Omnivorous | Benthic | 13 | Santos et al. (2020); Quintela et al. (2024); Freitas et al. (2025); Costa et al.(2022); Matos et al.(2022); Brito et al. (2024); Moraes et al. (2023); Custódio et al. (2020); Vasconcelos et al. (2021); Silva et al (2020); Gomes et al. (2020); Vargas et al (2024): Souza et al. (2025). |
| *Pygocentrus* ssp.  (Piranha) | Carnivore/  piscivore | Pelagic | 10 | Albuquerque et al. (2020); Waichman et al. (2025); Quintela et al. (2024); Freitas et al. (2025); Costa et al.(2022); Matos et al. (2022); Custódio et al. (2020); Moraes et al. (2023); Nyholt et al. (2022); Gomes et al. (2020). |
| *Leporinus* spp.  (Piau/Aracu) | Omnivorous | Bentopelagic | 9 | Santos et al. (2020); Quintela et al. (2024); Lacerda et al. (2020); Viana et al. (2022); Costa et al.(2022); Da Silva e Lima (2020); Vasconcelos et al. (2021); Gomes et al. (2020); Lima et al. (2025). |
| *Plagioscion squamosissimus*  (Pescada Branca) | Carnivore/  piscivore | Bentopelagic | 7 | Viana et al. (2022); Paiva et al (2024); Custódio et al. (2020); Azevedo-Silva et al. (2023); Da Silva e Lima (2020); Moraes et al. (2023); Costa et al.(2022). |
| *Pseudoplatystoma* spp*.*  (Surubim) | Carnivore/  piscivore | Bentopelagic | 5 | Meschede et al. (2024); Waichman et al. (2025); Silva e Lima (2020); Silva et al (2020); Vasconcelos et al. (2021). |
| *Hypostomus spp.*  (Cascudo) | Detritivore | Benthic | 5 | Viana et al. (2022); Lacerda et al. (2020); Freitas et al. (2025); Costa et al.(2022); Custódio et al. (2020);. |
| *Acestrorhynchus* ssp*.*  (Peixe-cachorro) | Carnivore/  piscivore | Pelagic | 5 | Viana et al. (2022); Brito et al. (2024); Azevedo-Silva et al. (2023); Cardoso et al (2025); Souza et al. (2025). |
